# Tibial morphology of symptomatic osteoarthritic knees varies according to location: a retrospective observational study in Japanese patients

**DOI:** 10.1038/s41598-024-53222-w

**Published:** 2024-02-08

**Authors:** Teruya Ishibashi, Shoji Konda, Masashi Tamaki, Seiji Okada, Tetsuya Tomita

**Affiliations:** 1 Department of Orthopaedic Biomaterial Science, Osaka University Graduate School of Medicine, 2-2 Yamadaoka, Suita, Osaka 565-0871 Japan; 2 Department of Health and Sport Sciences, Osaka University Graduate School of Medicine, 1-17 Machikaneyama, Toyonaka, Osaka 560-0043 Japan; 3 Department of Orthopaedic Surgery, Graduate School of Medicine, Osaka University, 2-2 Yamadaoka, Suita, Osaka 565-0871 Japan; 4 Graduate School of Health Sciences, Morinomiya University of Medical Sciences, 1-26-16 Nankokita, Suminoe-ku, Osaka-shi, Osaka 559-8611 Japan

**Keywords:** Rheumatic diseases, Osteoarthritis

## Abstract

This study analyzed 31 patients with symptomatic osteoarthritic knees scheduled to undergo knee arthroplasty or high tibial osteotomy and demonstrated shape variations in their proximal tibia using an average three-dimensional (3D) bone model. Preoperative computed tomography of the affected knees was reconstructed as 3D bone models using a triangle mesh of surface layers. The initial case was defined as the template, and the other models were reconstructed into homologous models with the same number of mesh vertices as that in the template. The corresponding mesh vertices of the other models were averaged to evaluate the spatial position on the particular mesh vertex of the template. This was applied to all the mesh vertices of the template to generate the average 3D model. To quantify the variation in surface geometry, average minimum distance from the average bone model to 31 models was recorded. The medial proximal tibial cortex (1.63 mm) revealed lesser variation compared to the tibial tuberosity (2.50 mm) and lateral cortex (2.38 mm), (*p* = 0.004 and *p* = 0.020, respectively). The medial tibial plateau (1.46 mm) revealed larger variation compared to the lateral tibial plateau (1.16 mm) (*p* = 0.044). Understanding 3D geometry could help in development of implants for arthroplasty and knee osteotomy.

## Introduction

Evaluations of bone shape and alignment are essential for understanding the pathology and progression of osteoarthritis (OA) of the knee^1–4^. Inappropriate sizing the femoral and tibial components in total knee arthroplasty (TKA) can be a risk for postoperative pain^5,6^. In addition, non-contoured plate in high tibial osteotomy (HTO) can increase concentrated stress, pain, and irritation^7,8^. Therefore, research on these topics assists in developing prostheses for TKA, HTO, and osteosynthesis^9–11^.

The morphological analyses of bone shapes using radiography and cadavers have been reported^12,13^. However, two-dimensional (2D) analyses are greatly affected by the projected direction of X-ray irradiation^14^. In addition, the challenges associated with cadaveric measurements are ethical considerations and the manual labor involved. Therefore, computed tomography (CT) and magnetic resonance imaging (MRI) are commonly used for bone morphological analysis in research^15–17^. These tools provide higher reproducible data that can be analyzed as three-dimensional (3D) bone shapes. Further, in 3D analysis, when the dimension is reduced to a 2D plane, such as coronal, axial, or sagittal, the evaluation value is greatly affected by the selected plane. In the last decade, several methods of 3D assessment of bone morphology have been reported in various medical fields^2,9,18–25^. In particular, statistical shape modeling is represented by methods such as 3D average model and principal component analysis (PCA) model^2,9,18–21,23–25^. Findings of these studies may be applied for bone substitutes (artificial bone) and the design of patient-specific instruments. Mahfouz et al. reported 3D morphological differences in the knee joints of the two sexes and among several races^9^. These findings could introduce various options for prostheses owing to the different sizes and shapes of bone morphology^21^. The problem with previous reports, however, is that quantitative comparison of surface geometry was lacking. In addition, previous studies have only shown the 3D morphology in normal adults^9,21^. Thus, quantitative 3D analysis of bone surface geometry in patients with symptomatic conditions could clarify whether large or small shape differences exist at specific locations.

Herein, we investigated whether the proximal tibia of a symptomatic osteoarthritic knee demonstrates variation or uniformity at a specific location. To quantify the surface geometry, we developed an average 3D bone model and compared the minimum distance between this average model and individual patients’ bone models in the specific region of interest (ROI).

## Results

**Figure 1:**
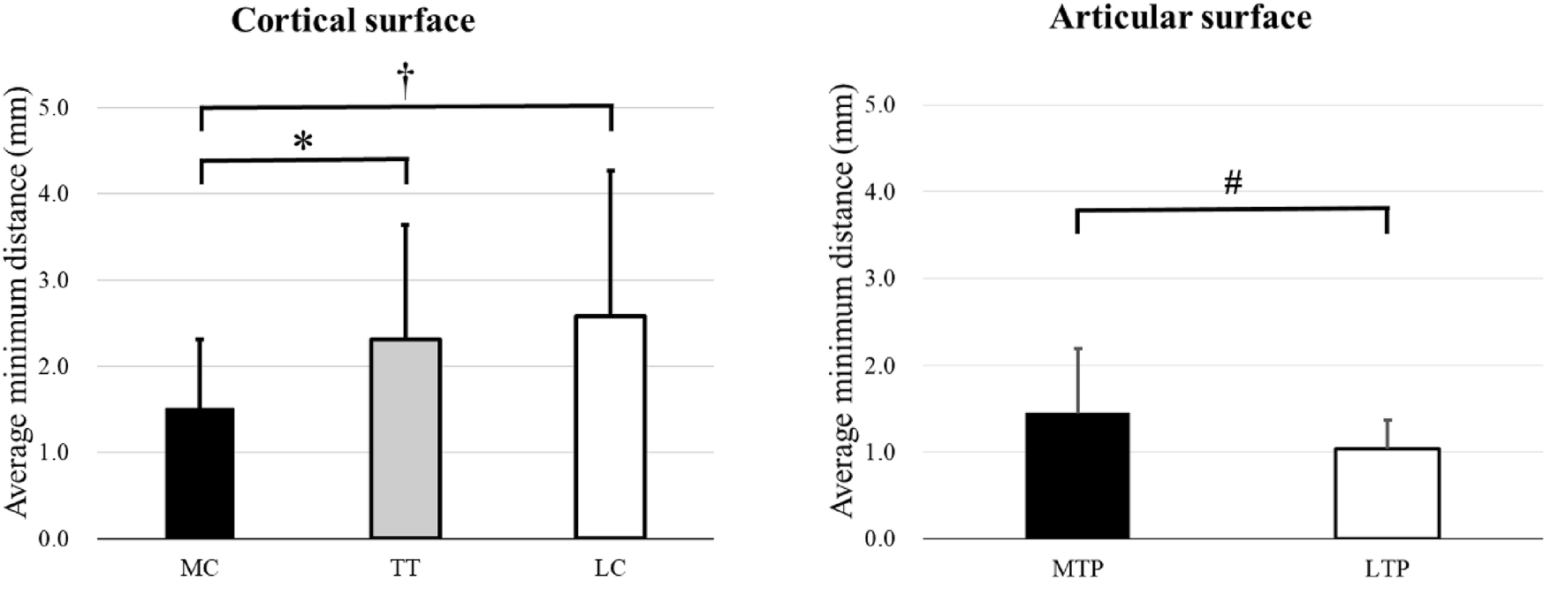
The average minimum distance from the average 3D bone model and individual 3D bone models in the specific region of interest (ROI) in the proximal tibial cortical surface and articular surface. † Indicates the significant difference between ROI-medial cortex (MC) and ROI-lateral cortex (LC). * Indicates the significant difference between ROI-MC and ROI-tibial tuberosity (TT). # Indicates the significant difference between ROI-medial tibial plateau (MTP) and ROI-lateral tibial plateau (LTP).

**Figure 2:**
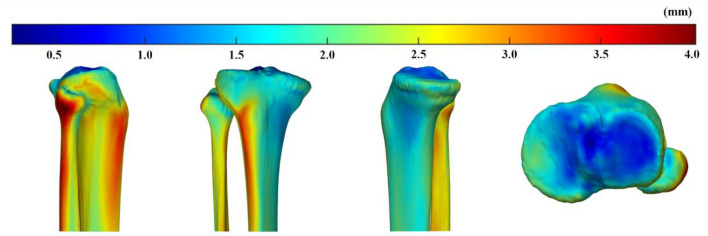
Color map demonstrating the minimum distance from the average 3D bone model to 31 individual bone models in all the mesh vertices. In the extra-articular bone surface, the medial side showed smaller minimum distances than the lateral side. On proximal articular surface, the lateral tibial plateau showed smaller minimum distances than the medial tibial plateau.

The average minimum distance in the ROI-medial cortex (MC) (1.63 mm [standard deviation (SD): 0.78 mm]) was less than that in the ROI-tibial tuberosity (TT) (2.38 mm [SD 1.31 mm]; 95% confidence interval [CI] for the difference: 0.34 mm, 1.18 mm; p = 0.004) and ROI-lateral cortex (LC) (2.50 mm [SD 1.69 mm]; 95% CI for the difference: 0.25 mm, 1.50 mm]; *p* = 0.020) (Figs. 1 and 2). The average minimum distance in ROI-medial tibial plateau (MTP) was larger than that in ROI-lateral tibial plateau (LTP) (1.46 mm [SD 0.75 mm] in ROI-MTP, compared with 1.16 mm [SD 0.49 mm] in ROI-LTP; 95% CI for the difference: 0.01 mm, 0.59 mm; *p* = 0.044) (Figs. 1 and 2).

## Discussion

The most important findings of this study were as follows. The average 3D bone models of 31 symptomatic osteoarthritic knees demonstrated variability and uniformity in specific locations. The MC surface had less variability compared with the TT and LC. In contrast, LTP exhibited less shape variety than the MTP in the proximal tibial articular surface. This novel method of evaluation of the 3D bone morphology could be utilized to quantify the surface geometry with different ROI selected by the assessors. It enabled accurate assessment of subtle localized variations encouraging the potential to develop implants for arthroplasty, osteotomy, and osteosynthesis.

**Figure 3:**
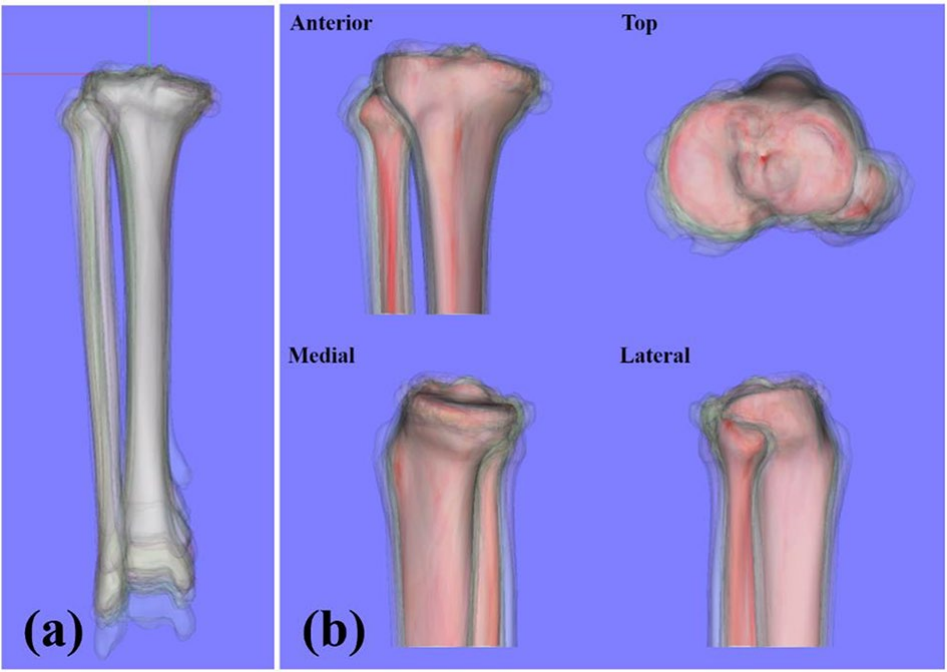
(**a**) Thirty one individual 3D bone models realigned in the tibial local coordinate system. (**b**) The average 3D bone model (red bone) and 31 individual 3D bone models (translucent bones).

We hypothesized that the morphology of the proximal tibia might vary according to the specific site in the pathological conditions of medial OA. In particular, medial OA revealed abnormalities of the medial femorotibial compartment, which naturally led to the expected result that a large variability would exist on the medial side. However, this study’s results were partially different from the hypothesis. In the present method, the morphology was expressed by obtaining the minimum distance between the average 3D bone model and each of the 3D bone models. Therefore, we could not potentially detect whether these subtle variations were due to the size or shape of the 3D bone model. When the tibial insertion of the anterior cruciate ligament (ACL) was used as the origin, the variation in the MC was less than those in the anterior and lateral cortices. However, there were various longitudinal length variations as seen in Fig. 3a. In other words, we obtained a uniform medial width in the metaphysis with minor variation in shape regardless of the longitudinal length. Conversely, the minimum distance on the lateral side was larger than that on the medial side. This might be ascribed to the possibility that the lateral width might be longer according to the longitudinal length. In other words, tibial size variations might be asymmetrical, not proportional, suggesting that the medial side was constant while the lateral side was variable. Another explanation was that greater variations in shape existed on the lateral side. Previous reports have shown that the proximal tibial torsion and varus change were associated with OA progression, and these deformities often occurred in the proximal metaphysis, although we could not detect if this was the cause or effect of OA^26–29^. In our study, tibial tuberosity and lateral cortex revealed shape variation. These results were consistent with those of previous studies of OA progression at the metaphysis^28,29^.

The TT has been widely used as a landmark of the rotation in the proximal tibia^30,31^. This landmark is clinically useful for the intraoperative assessment of alignment. However, several reports have revealed that the TT is unreliable as a landmark of the tibial rotational alignment^32–34^. An explanation of the lower reliability is that the size and position of the TT vary widely. In this study, the TT demonstrated variability in shape (the average minimum distance > 2 mm). Therefore, using the TT as a landmark has a potential risk of misleading the AP axis and axial rotation.

Regarding the proximal articular surface, the selected ROIs did not contain the peripheral region. This prevented the effect of osteophytes on the morphological analysis in this study. With OA progression, articular cartilage wear and bone erosion occur in the anteromedial tibial plateau and expand posteriorly and laterally^35,36^. In this study, the average minimum distance of the ROI-MTP was larger than that of the ROI-LTP. This study included patients from early to end-stage OA. Therefore, the surface erosion of MTP differed to some extent and resulted in substantial variability of MTP. In contrast, valgus OA was not included in this study; thus, the lateral femorotibial compartment might not be affected, resulting in less LTP variability. In a future study, we plan to analyze the valgus OA.

Since previous 2D evaluation methods for bone morphology had potential limitations in reliability and accuracy^13,14^. 3D evaluation methods have been reported in recent years; however, these methods evaluated lengths and angles in 2D planes, making it difficult to compare 3D parameters such as surface geometry^10,15–17,22^. Therefore, bone morphology based on PCA has been used in several fields^2,9,18–21,23–25^. In this study, the technique used to create the average 3D model was similar to those from previous reports, but the minimum distance from the average 3D bone model relative to each 3D bone model was used to quantify surface geometry. In conventional PCA, the first component (PC mode #1) reflected the variation of the tibia length, and its contribution rate was substantially high^23–25^. When averaging the tibia length, statistical shape models using PCA may lead to underestimating rotational deformity and width variations.

One of the strengths of this study is that it quantified surface bone geometry, not by averaging the length of the tibia but by using the minimum distance from the average model to the individual models. Moreover, the ROIs used in this study were chosen based on the clinical setting. As a highly reproducible local coordinate system (LCS) was set, any ROIs could be used to evaluate bone morphology quantitatively. Using this parameter, we can detect the region for better fitting the plate for HTO and proximal tibial fractures. In addition, this parameter could be an important factor for considering the alignment of tibial component and for developing new prosthesis in which the stem and keel of the tibial component do not come in contact with the proximal tibial cortex.

There were potential limitations in this study. First, this study only included the Japanese population as we sought to address whether variations exist in specific positions in the 3D bone morphology of the proximal tibia in symptomatic OA. Therefore, future studies are needed to detect the difference in morphology among different ethnic groups. Second, we included symptomatic OA patients. In this study, the hip-knee-ankle angle (HKAa) indicated a relatively narrow SD (4.4°). Therefore, it provided meaningful information for future studies, which include investigating the 3D morphology according to disease severity, such as normal knees, severe varus deformities, and valgus deformities. Lastly, since the 3D bone models were created from CT, we could not detect the articular cartilage and meniscus in the proximal tibial articular surface. In this study, however, CT rather than MRI was used to focus on bone surface geometry^37,38^. Therefore, the bone model created by CT was suitable for evaluating the bone cortex in this study.

In conclusion, the proximal tibial bone shape exhibited uniformity and variety in specific locations in symptomatic OA of the knee. The MC revealed less variability, whereas the TT and LC revealed more variability. In the proximal articular surface, LTP showed less variability compared with the MTP. A new evaluation method for 3D bone shapes would facilitate gaining more insight into the features of symptomatic OA and developing the implants for arthroplasty, osteotomy, and osteosynthesis.

## Methods

### Study design and participants

This was a retrospective, observational study approved by Ethical Review Board of Osaka University Hospital (approval number 13106). The following methods were carried out in accordance with the relevant guidelines and regulations. All patients provided written informed consent. The study was conducted at a single university hospital. The inclusion criteria were as follows: patients had symptomatic OA and were scheduled to undergo TKA, uni-compartmental arthroplasty, or HTO at our institution from October 2018 to October 2019. The exclusion criteria included patients with osteonecrosis of the knee, valgus knee OA, a history of fractures and infections involving the knee joint, and who underwent revision surgery. A total of 31 patients of Japanese origin were included. Patient characteristics are summarized in Table 1. Long leg standing (AP) and short lateral X-rays were conducted for preoperative evaluation. The HKAa measures the angle between the femoral and tibial mechanical axes. Varus is denoted as appositive and valgus as negative. The posterior tibial slope shows the angle between the tangent line of the MTP and the line perpendicular to the proximal tibial anatomical axis^39^.

**Table 1:** Patients’ demographic characteristics.

	N = 31
Age* (years)	72.2 (7.3; 53–84)
Sex (female/male)	22/9
Height* (cm)	154.6 (8.1; 140.9–173.0)
Weight* (kg)	62.6 (11.3; 44.8–89.7)
HKAa* (°)	10.9 (4.4; − 0.8 to 18.9)
PTS* (°)	7.4 (3.4; 1.0–15.3)

*Values are expressed as the average (standard deviation; range).

OA, osteoarthritis; HKAa, hip-knee-ankle angle; PTS, posterior tibial slope.

### Creating 3D bone models

All patients routinely underwent CT scans for preoperative evaluation. The CT data were obtained using a CT scanner (Optima CT660Pro Advance; GE Healthcare, Milwaukee, USA), and the scan was conducted under the following conditions: slice thickness (1.25 mm), tube voltage (120 kV), tube current (440 mA), and acquisition matrix (512 × 512). 3D bone models of the tibia and fibula were semi-automatically reconstructed using a commercially available computer software (Synapse Vincent; Fuji Medical Systems, Tokyo, Japan).

### Local coordinate system

**Figure 4:**
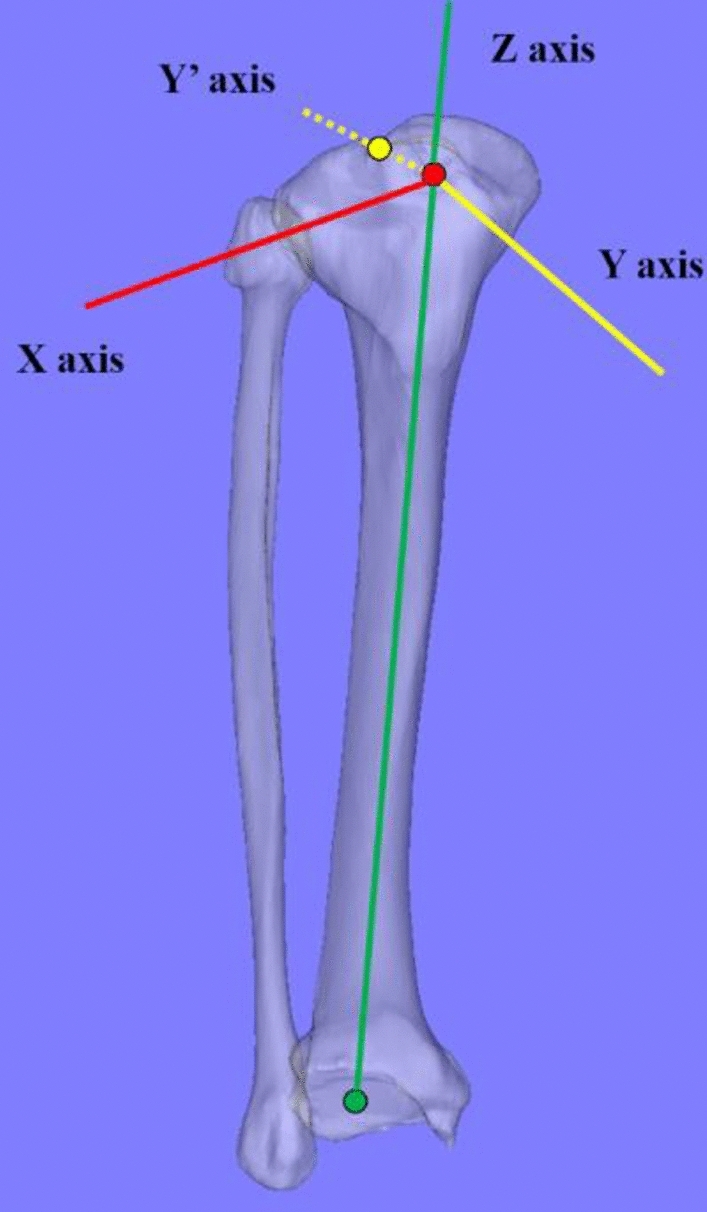
Definition of the tibial local coordinate system. Reference points consisted of the tibial insertion of the anterior cruciate ligament (ACL) (red dot), the posterior cruciate ligament (PCL) (yellow dot), and the centroid of the tibial plafond (green dot). The tibial insertion of the ACL was defined as the origin. Z axis (solid green line) passed through the origin and the centroid of the tibial plafond. The Y axis (yellow dotted line) passed through the origin and the tibial insertion of the PCL. The X axis (solid red line) was defined as the line perpendicular to the Y and Z axes. Y axis (solid yellow line) was defined as the line perpendicular to the X and Z axes.

We defined the tibial LCS, as indicated below, using a commercially available software program (Bone Simulator; Orthree, Osaka, Japan). First, the tibial insertion of ACL was defined as the origin^40^. Second, the Z axis passed through the origin and the centroid of the tibial plafond. The Y’ axis passed through the origin and the tibial insertion of the posterior cruciate ligament^41^. The line perpendicular to the Y’ and Z axis was established as the X axis. Finally, the line perpendicular to the X and Z axis was determined as the Y axis (Fig. 4). The intraclass correlation coefficient of the translation and rotation of the LCS revealed an almost perfect agreement and substantial agreement in the intra- and inter-rater reliability, respectively (Table 2)^42^. Thirty-one individual 3D bone models were realigned based on this LCS and used to generate the average 3D bone model (Fig. 3). All tibias and fibulas were converted to the right side in the mirror image if the affected bones were on the left side. Thereafter, the 3D bone models were realigned in the tibial LCS (Fig. 3a). To evaluate the 3D morphology of the proximal tibia, the 3D bone models were cut 13 cm distal from the origin (Fig. 3b).

**Table 2:** The intraclass correlation coefficient of the tibial local coordinate system.

	Translation (°)			Rotation (°)		
	X	Y	Z	X	Y	Z
Intra-rater reliability	0.999	0.987	0.999	0.996	0.989	0.999
Inter-rater reliability	0.999	0.978	0.999	0.999	0.978	0.737

### Creating the average 3D bone model

First, the patient’s initial 3D bone model was created as the template. The other 3D bone models were converted to homologous 3D bone models, with the same number of mesh vertices as those of the template model.

To create homologous 3D bone models, the template model was registered to other models by a non- liner free-form deformation algorism using the Image Registration Tool Kit^43^. Next, we measured the 3D distance from one mesh vertex on the template model to the corresponding mesh vertices on the other 3D bone models. Following the acquisition of the average 3D distance on the particular mesh vertex of the template model, the other mesh vertices on the template were evaluated similarly to obtain the average distance on all the mesh vertices. Based on the average 3D distances, an average 3D bone model was reconstructed (Fig. 3b).

### Region of interest

**Figure 5:**
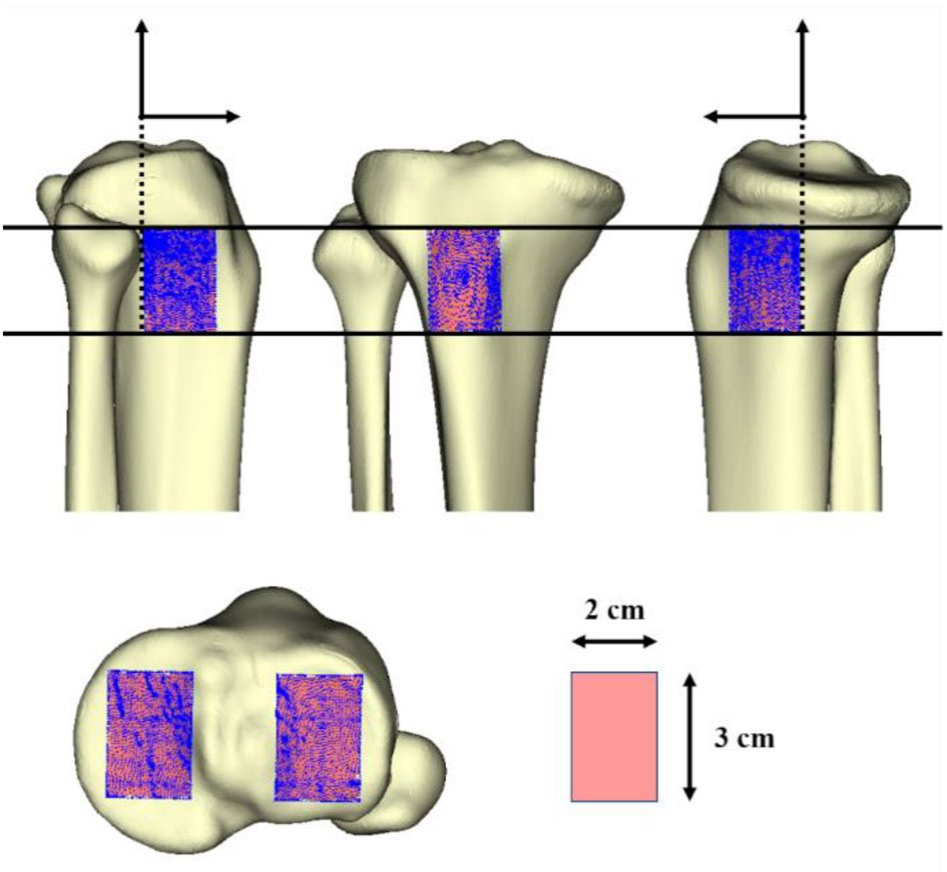
The definition of the region of interest (ROI) in the proximal tibial cortex surface and articular surface. The average 3D bone model detected the tibial tuberosity, and the area was approximately 2 cm wide and 3 cm long. The medial and lateral proximal tibial cortexes were cut by the XY plane (same height as the tibial tuberosity9 and the XZ plane (Y: 0/+ 2 cm) and defined as the ROI-medial cortex and ROI-lateral cortex, respectively. In the proximal tibial articular surface, the midpoints of anteroposterior tibial articular edges were detected on the medial (black dot) and lateral (black arrowhead) tibial plateau, respectively. Based on each midpoint, the XZ plane (Y: ± 1.5 cm) and YZ plane (X: ± 1.0 cm) cut each articular surface and defined these as ROI-medial tibial plateau and ROI-lateral tibial plateau, respectively. Blue dots in each ROI exhibited mesh vertices (including at least 1422 mesh vertices).

In the clinical setting, the TT is often used as a landmark of the AP axis, axial rotation, and osteotomy position for HTO^30,31,44^. We determined the TT of the average 3D bone model and the area of TT as approximately 2 cm wide and 3 cm long (Fig. 5). The ROI was defined in the proximal tibial cortex relative to the TT and proximal tibial articular surface as follows: first, we defined the surface of the TT as ROI-TT. Second, the medial and lateral tibial cortices were cut by the XY planes (same height as the ROI-TT) and the XZ plane (Y: 0/+ 2 cm) and defined this as the ROI-MC and ROI-LC, respectively (Fig. 5). The midpoint of AP articular edges was detected on the MTP and LTP in the proximal tibial articular surface. Based on each of the midpoints, the XZ (Y: ± 1.5 cm) and YZ (X: ± 1.0 cm) planes cut each articular surface and defined the areas as ROI-MTP and ROI-LTP, respectively (Fig. 5). These ROIs were 3D surfaces cut by 2 × 3 cm planes, and the surface areas although different, included enough mesh vertices to measure the distance.

### Measurements

**Figure 6:**
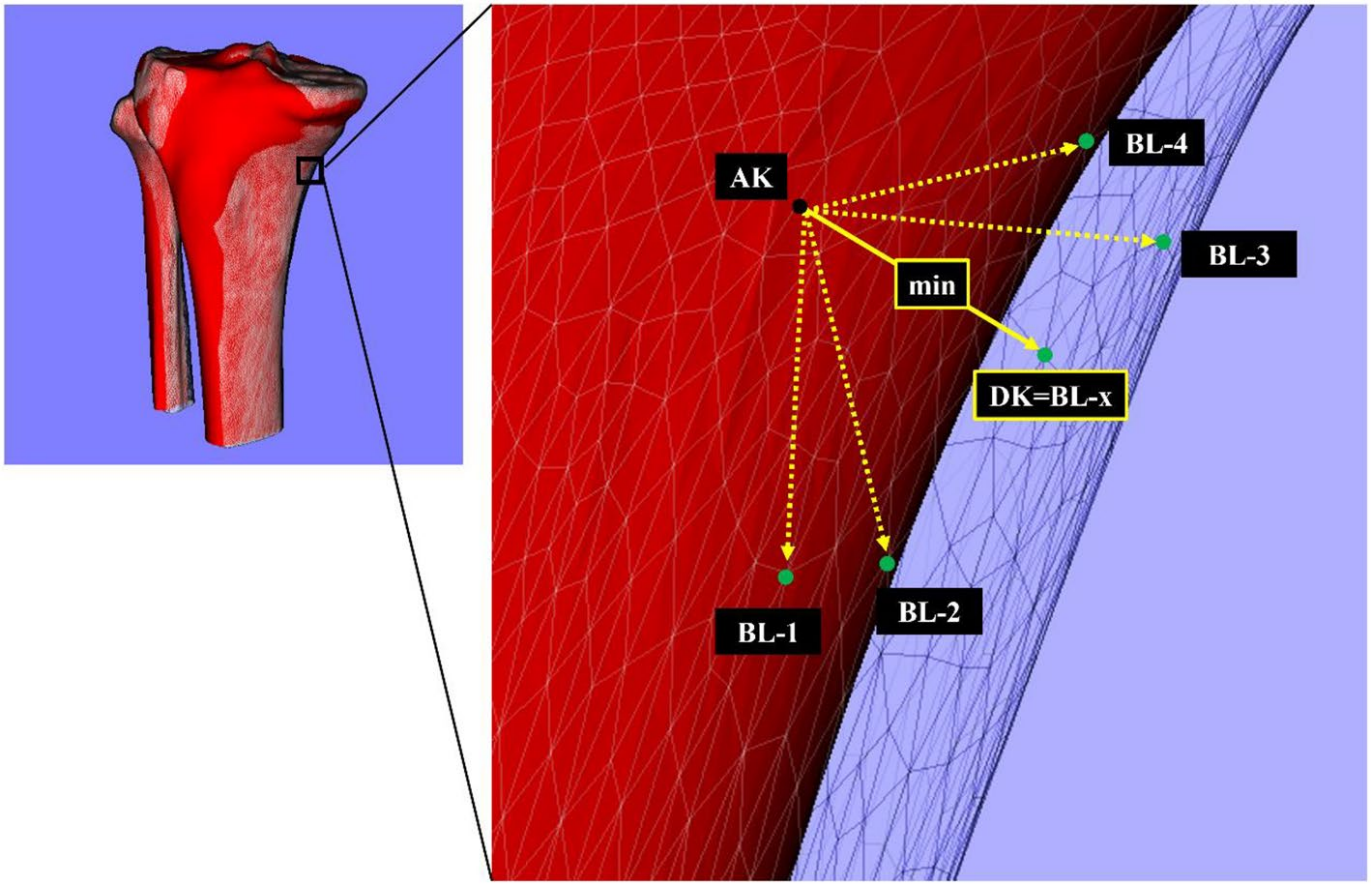
The minimum distance (DK) from the particular mesh vertex (AK) of the average 3D bone model to the mesh vertices (BL-1, BL-2, BL-3, …, BL-N) of the individual 3D bone model (BL = B1, B2, B3, …, B31). Black (AK) and green dots (BL-1, BL-2, BL-3, …, BL-N) indicate the particular mesh vertex of the average 3D bone model and candidate mesh vertices of the individual 3D bone models, respectively. Yellow dotted arrows indicate the candidates of minimum distance. The yellow solid arrow indicates the minimum distance (DK) on this mesh vertex of the average 3D bone model. The minimum distance was automatically detected and applied to all mesh vertices of the average 3D bone model (N = 54,764 mesh vertices).

The minimum distance was calculated from a particular mesh vertex (AK) of the average 3D bone model to the closest mesh vertex (DK) on individual bone model (BL) (Fig. 6). The minimum distance in all mesh vertices (AK = A1, A2, A3, …, An) of the average 3D bone model was evaluated. Next, the minimum distance of all bone models (BL = B1, B2, B3, …, B31) was evaluated. Finally, these minimum distances in the specific ROI were averaged.

### Statistical analysis

Two investigators measured the translation and rotation of the LCS in a blinded manner. One of the investigators measured them at a 3-week interval. To test intra- and inter-rater reliability, the intraclass correlation coefficient was calculated by two investigators. We analyzed the average minimum distance within the ROI of the proximal tibial cortex surface and the proximal tibial articular surface using repeated measures analysis of variance with Bonferroni corrected *t*-tests and the paired *t*-test. All data were expressed as means with standard deviations (SDs). Statistical significance was set at a *p* ≤ 0.05. Based on the preliminary ten cases, we obtained an SD of 0.5 mm for the average minimum distance in the ROI. Our calculations showed that a minimum sample size of 24 pairs of participants would be required to detect a difference of 0.3 mm for paired t-tests, with a type I error of 5% and 80% power. Statistical analysis was performed using JMP 13 (SAS Institute Inc, Cary, NC, USA) and EZR (Saitama Medical Centre, Jichi Medical University, Saitama, Japan)^45^.

## Data Availability

The data that support the findings of this study are available from the corresponding author on reasonable request.
